# From Mechanism to Manufacture: A Narrative Review of Insole Interventions Across Foot Deformity, Limb-Length Discrepancy, and Paediatric Orthopaedic Disease

**DOI:** 10.3390/bioengineering13101117

**Published:** 2026-09-24

**Authors:** Se Yeong Jeon, Chul Hee Jung, Seok Yeon Choi, Dong Ha Lee

**Affiliations:** 1 Science and Technology Department, Ulsan National Institute of Science and Technology, Ulsan 44919, Republic of Korea; skiende74@gmail.com; 2 Department of Orthopaedic Surgery, Aerospace Medical Center, Republic of Korea Air Force, Cheongju 28187, Republic of Korea; 3 Department of Rehabilitation Medicine, Aerospace Medical Center, Republic of Korea Air Force, Cheongju 28187, Republic of Korea; 4 Department of Orthopaedic Surgery, Seoul National University Hospital, Seoul 03080, Republic of Korea

**Keywords:** foot orthosis, insole, plantar pressure, finite element analysis, additive manufacturing, flatfoot, pes cavus, limb-length discrepancy, paediatric orthopaedics, smart insole

## Abstract

Insoles are among the most widely prescribed devices in orthopaedic practice, spanning conditions as mechanically dissimilar as paediatric flexible flatfoot, painful pes cavus, diabetic neuropathy, inflammatory arthritis, medial knee osteoarthritis, limb-length discrepancy, and neuromuscular gait disorders. Despite this breadth, the field is characterised by a persistent and instructive paradox: the biomechanical effects of insoles are reproducible and quantifiable, whereas their symptomatic effects are inconsistent and frequently indistinguishable from sham. This narrative review, drawing on a bounded, non-systematic evidence base of 55 sources across ten conditions, argues that this paradox is at least partly an engineering and measurement problem rather than a purely clinical one. We develop a mechanistic taxonomy in which every insole acts through some combination of four pathways—plantar pressure redistribution, external joint moment modulation, soft-tissue strain reduction, and sensorimotor modulation—and map the controllable design parameters (arch support height, medial posting angle, heel cup depth, thickness, material stiffness, and lattice architecture) onto each pathway using sensitivity data from a small number of heterogeneous finite element and Taguchi-based optimisation studies, several of which are single-model or single-patient analyses with limited experimental validation. We then review the manufacturing pathway from morphological capture through computer-aided design, additive or subtractive fabrication, and in-shoe verification, and summarise the condition-specific clinical evidence across the ten indications. Three hypothesis-generating findings emerge, each requiring prospective confirmation before being treated as established. First, custom devices consistently modify mechanics more than prefabricated devices, yet consistently fail to outperform them on symptom endpoints, indicating that the mechanism–outcome link is mediated by unmeasured variables. Second, the biomechanical response is heterogeneous in several conditions—for example, roughly one-third of patients with medial knee osteoarthritis show an increase rather than a decrease in the knee adduction moment with a lateral wedge—raising the possibility that population-averaged trials may obscure responder-specific effects, although the one trial that acted on this logic by individually tailoring the wedge angle found no clinical benefit. Third, a secondary analysis of a single randomised trial in painful pes cavus found that the redistribution, rather than magnitude, of plantar pressure predicted pain relief; if replicated, this would suggest that peak plantar pressure is an incomplete outcome measure in that specific pain-related context, a claim we do not extend to conditions such as diabetic offloading, where peak local pressure remains a mechanistically appropriate proxy for a different, site-specific outcome. We propose a design-decision framework, presented explicitly as a falsifiable hypothesis rather than a validated protocol, that selects design parameters by target mechanism rather than by diagnostic label, and argue that closed-loop prescription—instrumented insoles providing the continuous verification of both mechanical target attainment and wear adherence—is a promising but as-yet-unproven direction for the field.

## 1. Introduction

Few orthopaedic devices are prescribed as widely, or evaluated as inconsistently, as the insole. The same class of device—a contoured interface placed between the plantar surface and the shoe—is used to relieve heel pain in a runner, to redistribute pressure away from a neuropathic ulcer, to shift load from the medial to the lateral tibiofemoral compartment, to compensate for a two-centimetre limb-length discrepancy, to modify the posture of a paediatric flatfoot, and to provide sensory input in a child with cerebral palsy. These applications share almost nothing clinically. They share a great deal mechanically.

This gap between clinical heterogeneity and mechanical commonality is, we contend, the reason the literature is so difficult to interpret. The reviews are organised by diagnosis, so that the plantar fasciitis literature and the flatfoot literature and the diabetic offloading literature develop in parallel with minimal cross-fertilisation, each rediscovering the same design principles and the same null results. A Cochrane review of custom-made foot orthoses for foot pain illustrates the fragmentation directly: eleven trials involving 1332 participants were included, but these comprised five trials in plantar fasciitis, three in rheumatoid arthritis, and one each in pes cavus, hallux valgus, and juvenile idiopathic arthritis [1]. Each subgroup was too small to support conclusions; yet, the underlying devices differed less than the diagnoses did.

The consequences are visible in the evidence itself. Foot orthoses reliably alter plantar pressure distribution and reliably alter external joint moments; these are measurable, reproducible physical effects. Yet, the symptomatic evidence is repeatedly equivocal. For plantar heel pain, a meta-analysis of nineteen trials comprising 1660 participants concluded that there was moderate-quality evidence for pain reduction in the medium term, while explicitly noting uncertainty as to whether the change was clinically important [2]. A more recent meta-analysis of eight randomised trials in 391 patients with plantar fasciitis found a significant pain effect but no significant difference in foot function or walking ability, and reported no adverse events [3]. For paediatric flexible pes planus, a systematic review concluded, after examining eleven studies, that uncertainty about effectiveness persists [4]. For medial knee osteoarthritis, lateral wedge insoles reduce the external knee adduction moment on average, but trials comparing them with neutral insoles have not demonstrated superior pain relief [5,6].

A field in which the mechanism works and the treatment does not have a measurement or targeting problem, does not have a mechanism problem. The purpose of this review is to make that argument explicitly and to organise the evidence in a way that supports it. We adopt an engineering rather than a diagnostic organisation: we begin from the mechanisms by which an insole can alter the mechanical state of the lower limb, map the controllable design parameters onto those mechanisms using quantitative sensitivity data, review the manufacturing pathway that translates a design into a device, and, only then, survey the condition-specific clinical evidence. Our scope deliberately spans foot deformity, limb-length discrepancy, and paediatric orthopaedic disease, because the mechanistic commonalities across these domains are the central point.

## 2. Materials and Methods

This is a narrative review and was not registered. Reporting was informed by the SANRA (Scale for the Assessment of Narrative Review Articles) domains of justification, statement of aims, description of the literature search, referencing, appraisal, and presentation of data. PRISMA was not applied, as it pertains to systematic reviews.

Screening and study selection were performed by one author (S.Y.J.) and checked by the senior author (D.H.L.); a registered protocol, dual independent screening, and a formal risk-of-bias instrument were not used. We note this explicitly because it bears on interpretation: with 55 sources distributed across ten clinical conditions plus mechanistic, manufacturing, and sensor-technology content, condition-specific coverage in this review (typically four to six clinical sources per condition) is narrower than a dedicated systematic review of a single indication would provide (commonly on the order of 14–24 studies), and section-level claims should be read as a cross-condition synthesis intended to motivate hypotheses, not as a substitute for condition-specific systematic review.

Search strategy. PubMed/MEDLINE, Scopus, Web of Science, and Google Scholar were searched for English-language records published between 1 January 1995 and 31 May 2026. Search strings combined device terms (insole, foot orthosis, foot orthotic, shoe insert, shoe lift, heel lift, and lateral wedge), mechanism, measurement, and design-optimisation terms (plantar pressure, knee adduction moment, plantar fascia strain, finite element, Taguchi method, response surface methodology, design of experiments, gait analysis, and centre of pressure), manufacturing terms (3D printing, additive manufacturing, CAD/CAM, lattice, and metamaterial, 3D scanning), and condition terms (pes planus, flatfoot, pes cavus, hallux valgus, plantar fasciitis, plantar heel pain, posterior tibial tendon dysfunction, progressive collapsing foot deformity, diabetic foot, rheumatoid arthritis, juvenile idiopathic arthritis, knee osteoarthritis, leg length discrepancy, limb length discrepancy, and cerebral palsy). Reference lists of retrieved reviews were hand-searched. The terms Taguchi method and response surface methodology were not part of the initial database strings; the finite element and design-optimisation literature discussed in Section 3.2 was identified largely through hand-searching the reference lists of retrieved biomechanics reviews and through citation snowballing from the pes cavus and diabetic offloading trials, and search completeness on these two terms specifically was confirmed with a supplementary targeted search on 31 May 2026. We flag this two-stage process as a limitation on the systematicity of the engineering-literature search (Section 8).

Selection. Priority was given, in descending order, as follows: systematic reviews and meta-analyses; randomised controlled trials with sham or active comparators; prospective biomechanical studies with instrumented gait or in-shoe pressure measurement; finite element and design-optimisation studies with stated parameterisation; and manufacturing or sensor-technology studies with reported performance metrics. Studies of ankle–foot orthoses were included where they informed mechanism or where insole and ankle–foot orthosis evidence is inseparable in a given condition, notably in cerebral palsy and progressive collapsing foot deformity. Case reports, narrative commentaries without primary data, and non-English records were excluded. Open-access sources were preferred to maximise the accessibility of the evidence base to clinicians and engineers without institutional subscriptions.

Because this review’s subject is the insole rather than the ankle–foot orthosis, evidence drawn from ankle–foot orthosis studies (Section 5.9, in particular) is used as indirect, mechanistically analogous evidence rather than as direct evidence for insoles, and mechanistic transfer between the two device classes has not been independently established; we flag this explicitly wherever such evidence is used.

Synthesis. Data were synthesised descriptively. No statistical pooling was attempted; the heterogeneity of device design, comparator choice, outcome measure, and follow-up duration across this literature makes meta-analysis inappropriate outside narrowly defined subgroups. Where quantitative sensitivity data from design-optimisation studies are reported, the parameterisation and loading condition are stated so that transferability can be judged.

This caveat applies with particular force to the finite element and Taguchi studies summarised in Table 1: these studies differ from one another in the design factors examined, the levels and ranges tested, boundary and loading conditions, material assumptions, and sample size, including at least one single-patient case study. The variance percentages reported by each study are specific to that study’s own parameterisation and are not statistically poolable or directly comparable across studies; they are presented side by side in Table 2 for qualitative, hypothesis-generating comparison only.

## 3. Mechanistic Framework

### 3.1. Four Pathways of Action

Every insole, regardless of indication, acts through some combination of four mechanisms. Separating them is useful because they respond to different design parameters and require different verification measurements.

Pressure redistribution. By increasing the contact area between the plantar surface and the support, a contoured device reduces the peak pressure at loaded regions and transfers the load to previously unloaded regions, principally the medial longitudinal arch. This is the dominant mechanism in diabetic offloading and in forefoot pathology.

External joint moment modulation. By altering the mediolateral position of the centre of pressure, a wedged device changes the moment arm of the ground reaction force about proximal joints. This is the mechanism invoked for lateral wedge insoles in medial knee osteoarthritis and for medial posting in pronation-related pathology.

Soft-tissue strain reduction. By supporting the medial longitudinal arch, a device reduces the elongation of the plantar aponeurosis and the tensile demand on the tibialis posterior tendon. This is the mechanism invoked in plantar fasciopathy and in progressive collapsing foot deformity.

Sensorimotor modulation. By altering the cutaneous plantar afferent input, textured or contoured surfaces may modify the muscle activation timing and postural control independently of any kinematic change. This mechanism is the least well characterised and is invoked most often in paediatric neuromuscular conditions.

Table 1 maps these mechanisms onto the design parameters that principally govern them and onto the measurement modality required to verify them.

### 3.2. Quantitative Design Sensitivity

The most useful contribution of computational modelling to this field has been to rank design parameters by their influence, which cannot be done experimentally without prohibitively large factorial trials. Combined finite element and Taguchi orthogonal-array studies have converged on a consistent hierarchy.

Two methodological qualifications apply to all of the studies discussed in this subsection and are stated once here rather than repeated at every citation. First, none of the cited finite element studies reports a mesh convergence analysis, and the reporting of the soft-tissue material models, contact conditions at the foot–orthosis interface, and experimental (cadaveric or in vivo) validation of model outputs is inconsistent and, in several cases, absent; one of the paediatric models discussed below is a single-patient (n = 1) case study. These are properties of the underlying primary literature rather than of this review, but they mean the variance percentages that follow should be read as indicative of a modelling result under stated assumptions rather than as validated physical measurements. Second, the Taguchi orthogonal-array method used in most of these studies is a fractional-factorial screening design that evaluates a small number of factor levels with substantially fewer experimental runs than a full factorial design, optimises a single response through a signal-to-noise ratio, and is comparatively insensitive to interaction effects between factors. This is not a hypothetical concern: a direct comparison of the Taguchi method against the response surface methodology on the same additive-manufacturing process found that the two approaches ranked the influence of process parameters differently and identified different optimal parameter combinations [6]. None of the studies discussed below pairs its Taguchi analysis with a response-surface or full-factorial interaction check, so the possibility that interaction effects between, for example, the arch height and material stiffness are under-represented in the reported rankings cannot be excluded from this literature alone.

In a foot-orthosis optimisation study using four design factors, a higher arch support accounted for 45.7% and medial posting inclination for 25.5% of the variance in peak plantar pressure; for proximal plantar fascia strain, arch support accounted for 41.8% and material stiffness for 37%, with the important qualification that a higher arch support combined with softer material minimised the peak fascia strain [7]. A comparable pattern was also observed in a separate muscle-driven finite element flatfoot model during walking rather than a static stance, in which plantar pressure and plantar fascia loading were both sensitive principally to the arch support feature [8]; the two models differ in loading condition and are not a replication of one another in the strict sense.

Earlier work using a combined finite element and Taguchi approach across five design factors—arch type, insole thickness, midsole thickness, insole stiffness, and midsole stiffness—found a custom-moulded shape to be the single most important factor in reducing peak plantar pressure, with insole stiffness second [9]. Parametric finite element analysis across six insole materials spanning elastic moduli from 100 MPa to 500,000 MPa under a static bilateral stance and forefoot contact has since provided a quantitative basis for material selection, an area the authors noted had previously relied largely on empirical or trial-and-error approaches [10].

We then apply the same method to a paediatric case, a patient-specific model of a child with hereditary spastic paraplegia, pes planovalgus, and flexed-knee gait evaluated arch height, heel cup depth, thickness, and material across a Taguchi L9 array under a vertical ground reaction force of 1.1 times body weight [11]. The optimal configuration—42 mm arch height, 20 mm heel cup depth, 10 mm thickness, ethylene-vinyl acetate—achieved a peak plantar pressure of 0.087 MPa. Arch height dominated plantar pressure (79.4% of variance), deformation (68.1%), and strain (48.2%), whereas heel cup depth was most influential for stress (40.2%) and material type contributed minimally to pressure and deformation while exerting a greater effect on stress (11.6%) and strain (15.0%); because this is a single-patient (n = 1) model, these figures describe the optimum for one child’s geometry and loading condition rather than a generalisable population parameter.

Table 2 summarises this evidence. Within the specific parameter ranges, boundary conditions, and single-response optimisation criteria used in each cited model, the arch support geometry consistently accounts for the largest share of the reported variance in plantar pressure and fascia strain, with material stiffness contributing more to internal tissue stress than to surface pressure. We present this as a consistent pattern across a small number of heterogeneous, model-dependent studies rather than as a generalisable biomechanical principle or as evidence of comparable clinical effect sizes: the studies differ in the factors tested, parameter ranges, mesh and material assumptions, and sample size (Section 3.2), their variance percentages are not directly comparable to one another, and none has been externally validated against an independent experimental or clinical dataset. Read cautiously, the pattern nonetheless has a practical implication worth testing further: within an individual design-optimisation exercise of this kind, arch geometry may warrant attention at least as early as material selection, and prescriptions that specify the material durometer without specifying the arch geometry may be optimising a secondary parameter relative to a primary one—a hypothesis this review’s evidence base can motivate but not confirm.

**Table 2:** Reported design-parameter sensitivity from finite element and Taguchi optimisation studies. The studies differ in design factors, parameter ranges, boundary conditions, and sample size—the paediatric HSP study (final three rows) is a single-patient case report—and the variance percentages are study-specific; they are not statistically poolable across rows and should not be read as a quantitative meta-analysis.

Study Context	Outcome	Dominant Parameter (% Variance)	Secondary Parameter	Ref
Foot orthosis optimisation	Peak plantar pressure	Arch support (45.7%)	Medial inclination (25.5%)	[7]
Foot orthosis optimisation	Proximal plantar fascia strain	Arch support (41.8%)	Material stiffness (37%)	[7]
Muscle-driven flatfoot model, walking	Plantar pressure, fascia loading	Arch support	—	[8]
Five-factor orthosis model	Peak plantar pressure	Custom-moulded shape	Insole stiffness	[9]
Paediatric HSP, Taguchi L9	Peak plantar pressure	Arch height (79.4%)	Thickness	[11]
Paediatric HSP, Taguchi L9	von Mises stress	Heel cup depth (40.2%)	Material (11.6%)	[11]
Paediatric HSP, Taguchi L9	Strain	Arch height (48.2%)	Thickness (22.3%)	[11]

## 4. Design and Manufacturing Pathway

### 4.1. Morphological Capture

The traditional pathway begins with plaster casting or a foam impression box. Digital capture by three-dimensional surface scanning has largely displaced these in high-volume settings. A systematic review comparing 3D scanning with traditional capture methods for the fabrication of ankle–foot and in-shoe orthoses found that scanning combined with computer-aided manufacturing produced comparable devices within similar time frames, and noted that low-cost handheld scanners could outperform substantially more expensive units on capture speed [12]. The practical consequence is that capture is no longer a rate-limiting or cost-limiting step, which shifts the bottleneck downstream to design and verification.

### 4.2. Computer-Aided Design and Optimisation

Digital capture enables parametric design, and parametric design enables optimisation. The finite element studies described in Section 3.2 are the design-side expression of this capability: rather than fabricating and testing devices iteratively in wear trials, the design space can be explored computationally and only the optimum fabricated [7,8,9,10,11]. Optimisation frameworks have been extended to lattice architectures, in which local cell dimensions produce spatially varying stiffness within a single monolithic device, with numerical homogenisation used to make the optimisation computationally tractable [13].

This capability is genuinely new. A conventional device has a small number of discrete material zones set by what can be glued together; a lattice device has a continuously variable stiffness field that can be targeted region by region against a measured pressure map. The design freedom substantially exceeds what current prescription conventions are able to specify.

### 4.3. Fabrication

Additive manufacturing and computer-numerical-control milling are both mature for this application. Reviews of additive manufacturing in orthotic practice have catalogued the process routes, material selections, and workflow requirements, while noting that the principal barriers to clinical implementation are process qualification and clinician skill rather than device performance [14,15]. The reported material choices centre on ethylene-vinyl acetate, thermoplastic polyurethane, and polypropylene, with thermoplastic polyurethane favoured for lattice structures because of its elastomeric recovery (Table 3).

Comfort—the outcome that ultimately governs adherence—has been evaluated directly. A novel three-dimensionally printed foot orthosis incorporating variable-hardness meta-structures was compared for comfort against factory-made shoe insoles and traditional custom orthoses, addressing the concern that digitally produced devices might not be tolerated despite equivalent mechanical performance [16].

### 4.4. In-Shoe Verification and Instrumented Insoles

The step most often omitted is verification that the fabricated device achieved its mechanical target in the patient’s own shoe during the patient’s own gait. In-shoe pressure measurement systems make this feasible, and the diabetic literature has demonstrated its value: a systematic review with meta-analysis of footwear and insole design features found that pressure-informed design produced a peak pressure reduction of 75.4 kPa (95% CI −127.4 to −23.44; *p* < 0.004), substantially exceeding the reductions attributable to arch profile alone (37.5 kPa) or metatarsal addition alone (35.96 kPa) [17]. Designing from a measured pressure map roughly doubles the achievable offloading relative to designing from foot shape alone.

Instrumented or “smart” insoles extend verification from a single clinic measurement to continuous monitoring. Sensing modalities include resistive, capacitive, inductive, piezoelectric, triboelectric, and optical approaches, and a review of the field has identified insufficient sensor calibration, gait-based verification, and human-study validation as the principal weaknesses, proposing testing machines and instrumented treadmills as a reference standard [18]. Reported device performance is now adequate for clinical use: a three-dimensionally printed capacitive insole demonstrated a sensitivity of 1.19 MPa^−1^ across a working range below 872.4 kPa, linearity of R^2^ = 0.993, response and recovery times of 142–160 ms, hysteresis below 10%, and stability over at least 2280 cycles [19]. Comparative work on sensor selection has examined the complementary roles of force-sensitive resistors and piezoelectric elements for static and dynamic components of the pressure signal, respectively [20].

The significance for this review is that instrumented insoles measure both of the variables that we argue mediate the mechanism–outcome gap: whether the intended mechanical change was achieved, and whether the device was actually worn.

## 5. Condition-Specific Evidence

### 5.1. Plantar Heel Pain and Plantar Fasciopathy

This is the largest evidence base and the most equivocal. A meta-analysis of nineteen trials with 1660 participants found very low-quality evidence of no short-term effect and moderate-quality evidence of medium-term pain reduction, with explicit uncertainty as to clinical importance [2]. A separate systematic review examined twenty studies investigating eight distinct orthosis types, a heterogeneity that itself limits synthesis [21]. A 2025 meta-analysis of eight randomised trials in 391 patients confirmed a significant pain effect (*p* = 0.0003) with no significant effect on foot function (*p* = 0.23) or walking ability (*p* = 0.44), and no reported adverse events [3].

The dissociation between pain and function within the same analyses is notable and is consistent with a device that alters tissue loading without restoring capability. Comparative work has repeatedly found prefabricated and custom devices equivalent, including a randomised trial of prefabricated versus customised orthoses fabricated from low-cost foam for non-complicated plantar fasciitis [22].

### 5.2. Paediatric Flexible Pes Planus

Paediatric flatfoot is the indication in which prescription volume most exceeds evidentiary support. A systematic review identified eleven studies—three randomised trials, two case-controls, five case series, and one single-case study—employing a diverse range of prefabricated and customised devices with heterogeneous outcomes, and concluded that uncertainty about effectiveness persists despite consistently positive directional findings across pain, foot posture, gait, function, and structural measures [4].

More recent meta-analysis has provided the first quantitative structural estimates. Pooling six studies with 297 participants, foot orthoses produced significant radiological improvement in older children, with a mean difference of −2.76° (95% CI −4.30 to −1.21; *p* = 0.0005) in the lateral talar–first metatarsal angle, −5.14° (95% CI −7.76 to −2.52; *p* = 0.0001) in the lateral talo-heel angle, and 1.79° (95% CI 0.88 to 2.69; *p* = 0.0001) in calcaneal pitch [23].

These are real but small structural changes, and their symptomatic significance in a population that is largely asymptomatic and in which the arch develops spontaneously remains the central unresolved question. Radiographic angle change of a few degrees is a structural, not a patient-centred, outcome, and none of the included studies has established a correlation between the magnitude of radiographic correction and validated measures of pain, function, or quality of life in this population; the clinical meaningfulness of the meta-analytic angle changes reported above should not be assumed and remains to be demonstrated. From a design perspective, the finite element evidence in Section 3.2 is directly applicable, with the qualifications noted there: arch support height is the dominant parameter in the cited models, and the softer-material-plus-higher-arch combination minimises fascia strain within those models [7,8].

### 5.3. Adult Flexible Pes Planus and Progressive Collapsing Foot Deformity

In adults, the same deformity carries a clearer symptomatic burden and a progressive natural history. A systematic review of the orthotic treatment for stages I and II posterior tibial tendon dysfunction examined five databases and addressed the specific question of whether orthotic management is effective at these stages [24]; a companion review of foot orthoses for adults with flexible pes planus covered the non-tendinopathic presentation [25]. Biomechanical work has clarified the mechanism: medially wedged and thin-flexible foot orthoses both reduced ankle pronation and arch flattening during unilateral drop-jump landings on level and valgus-inclined surfaces, with medially wedged devices additionally decreasing midfoot dorsiflexion [26].

Real-world adherence data are now available. In a retrospective cohort of custom foot orthosis users with progressive collapsing foot deformity, 80% reported symptom improvement and 56% wore the devices for more than six hours per day, with comfort positively associated with both wear time and magnitude of pain reduction [27]. The last finding is the important one: comfort predicted wear time, and wear time predicted benefit. Comfort is therefore not a secondary ergonomic consideration but a mediator of efficacy.

### 5.4. Pes Cavus and Neuromuscular Foot Deformity

Pes cavus provides the cleanest positive evidence in this review, and also its most instructive mechanistic finding. In a randomised, single-blind, sham-controlled trial, 154 participants with chronic musculoskeletal foot pain and bilateral cavus feet were allocated to custom foot orthoses (n = 75) or sham insoles (n = 79) [28]. Foot pain improved more with custom orthoses (difference 8.3 points; 95% CI 1.2 to 15.3; *p* = 0.022), as did function (9.5 points; 95% CI 2.9 to 16.1; *p* = 0.005) and physical functioning quality of life (7.0 points; 95% CI 1.9 to 12.1; *p* = 0.008). Plantar pressure improved across all foot regions (difference −3.0 N·s/cm^2^; 95% CI −3.7 to −2.4; *p* < 0.001). A Cochrane review of interventions for pes cavus identified this as the only trial fully meeting the inclusion criteria [29].

The mechanistic re-analysis of this dataset is, in our assessment, one of the more provocative single-trial findings in the insole literature, and it should be weighted accordingly: it derives from a secondary, hypothesis-generating analysis of one randomised trial and has not, to our knowledge, been independently replicated in another cohort or condition. Examining the relationship between mechanical change and symptomatic change, the investigators found no relationship between the change in plantar pressure magnitude and the change in symptoms in either group; instead, the redistribution of plantar pressure, quantified by a Dynamic Plantar Loading Index derived from the probability distribution of peak pressure over time, had a significant effect on pain relief (*p* = 0.001) [30,31]. Pes cavus feet had a significantly lower regression factor than healthy feet (0.30 versus 0.51; *p* < 10^−7^), and this index changed only in the active treatment group.

If this finding is replicated, it would suggest that the shape of the loading distribution, rather than its peak magnitude, is the therapeutic variable in painful pes cavus, and that peak plantar pressure—the outcome measure used almost universally across this literature—may be an incomplete measure of benefit in that specific condition. We state this as a hypothesis rather than an established conclusion, and we do not extend it to conditions such as diabetic ulceration (Section 5.6), where peak local pressure at a defined anatomical site is the mechanistically appropriate proxy for a different outcome—focal tissue breakdown—rather than for pain modulation; the two conditions may simply call for different outcome constructs rather than one interpretation of plantar pressure being correct and the other flawed. Whether a redistribution-type index adds predictive value alongside peak pressure in other pain-related conditions is, to our knowledge, untested.

### 5.5. Inflammatory Arthritis: Rheumatoid and Juvenile Idiopathic

The rheumatoid foot has a well-defined mechanical target: forefoot pressure concentration under subluxed metatarsal heads. A double-blind randomised controlled trial in eighty women with rheumatoid arthritis found that foot orthoses incorporating metatarsal and medial arch support reduced pain during walking and at rest bilaterally, and that the duration of insole use correlated with improvements in both pain and function [32]. A further randomised trial evaluated varying insole types combined with specialised footwear [33]. The dose–response relationship with wear time again positions adherence as a mediator rather than a nuisance variable.

In juvenile idiopathic arthritis, the evidence is more limited but includes randomised data. An early randomised trial evaluated the efficacy of custom foot orthotics for pain and functional status in affected children [34], and a multicentre randomised clinical trial subsequently compared customised preformed devices against a flat sham innersole with pain as the primary outcome followed to twelve months, alongside quality of life, foot and ankle disability, and swollen and tender joint counts [35,36]. We describe this comparator as a flat sham rather than as an inert control, consistent with the position taken in Section 8: a flat insole still changes the contact interface between the foot and the shoe and cannot be assumed to be mechanically inactive. The comparator problem—that no available sham fully isolates the treatment mechanism from non-specific device effects—pervades this literature and applies to this trial as it does to the pes cavus trial discussed in Section 5.4.

### 5.6. The Diabetic Neuropathic Foot

Diabetic offloading is the indication with the clearest mechanical objective and the most developed engineering response, because the endpoint (ulceration) is unambiguous and the mediating variable (plantar pressure) is directly measurable. We use peak, or local, plantar pressure as the primary construct in this section because, unlike the pain-related outcome discussed for pes cavus in Section 5.4, the diabetic endpoint of interest—focal skin and soft-tissue breakdown—has a well-established, site-specific mechanical threshold; peak pressure at the ulcer-prone site is therefore an appropriate proxy for this specific outcome even if it is not the primary mediator of pain relief in other foot conditions (Section 5.4 and Section 6.4).

The custom-versus-prefabricated question was addressed in a participant-blinded randomised controlled trial [37]. The custom functional insole was modestly more effective than the prefabricated device in reducing the forefoot pressure–time integral at issue (27% versus 22%) and remained more effective at the six-month follow-up (30% versus 24%; *p* = 0.001), but was significantly more expensive (£656 versus £554; *p* < 0.001). The authors concluded that the prefabricated device should be preferred where clinically appropriate on cost-effectiveness grounds. Two adherence findings from the same trial deserve emphasis: full compliance, defined as a minimum of seven hours per day on seven days per week, was reported by only 40% of participants, and 76% reported a minimum of five hours per day on five days per week.

The design-feature evidence is quantitative. The meta-analysis found peak plantar pressure reductions of 37.5 kPa attributable to the arch profile, 35.96 kPa to the metatarsal addition, and 75.4 kPa to the pressure-informed design, with apertures also effective [17].

Additive manufacturing has produced measurable gains over the standard of care. Custom accommodative insoles designed from the combined foot shape and in-shoe pressure data, then three-dimensionally printed with patient-specific lattice stiffness, demonstrated an approximately 14% reduction in peak plantar pressure in key regions relative to the milled ethylene-vinyl acetate standard of care in healthy participants, and both pressure-based and finite-element-based printed designs significantly outperformed the standard of care on the peak plantar pressure and pressure–time integral in the region of interest, with finite-element designs additionally reducing pressure in adjacent regions [38]. Complementary work has evaluated graded-stiffness offloading and the mechanical design and wear performance of printed insoles in older adults with diabetes [39,40].

### 5.7. Medial Knee Osteoarthritis: The Responder Problem

Lateral wedge insoles are the clearest case of a mechanism that works in a treatment that does not; the reasons are only partly understood, and, as discussed below, the one trial that tested this review’s central biomechanical-targeting logic directly did not find a clinical benefit.

Systematic reviews confirm the mechanical effect: lateral wedge insoles produce small reductions in the external knee adduction moment and the knee adduction angular impulse during walking in people with medial knee osteoarthritis, of the order of 6% per step, but appear ineffective at attenuating structural change in the population as a whole, and may be better suited to targeted use in biomechanical phenotypes associated with larger load reductions [5]. Dose-finding meta-analysis across wedge angles grouped as ≤5°, >5° to ≤9°, and >9° found that higher wedge angles did not produce greater reductions than lower ones, supporting the selection of the lowest effective angle after a prior biomechanical analysis [41].

The decisive evidence concerns heterogeneity. In an ancillary analysis of 70 participants from a randomised trial, 33% increased their external knee adduction moment under the lateral wedge condition while 67% decreased it, with coronal–plane ankle joint complex biomechanics distinguishing responders from non-responders [42]. Subsequent work has sought clinically accessible predictors of the biomechanical response using demographic, clinical, and walking-related variables against response thresholds of 2%, 6%, and 10% reduction [43].

A twelve-week randomised controlled trial in which the wedge angle was individually tailored between 2° and 10° following prior biomechanical analysis, against a neutral 0° insole, nonetheless found no significant between-group difference in pain (−12.5 mm; 95% CI −29.4 to 4.4), biomechanical parameters, or physical performance tests, with the exception of one KOOS subscale [44]. Tailoring the wedge angle, in other words, was insufficient to produce a clinical benefit in this trial. One interpretation, which we find plausible but cannot confirm from this trial alone, is that selecting patients whose ankle mechanics permit the load transfer to occur is also necessary; an equally consistent interpretation is that a verified mechanical correction of this kind does not reliably translate into a symptomatic benefit in this condition, regardless of patient selection. The trial cannot distinguish between these two interpretations, and we discuss the implications for this review’s proposed framework in Section 6.2 and Section 7.

This tension is, in miniature, the central interpretive difficulty this review confronts. When roughly one-third of a treatment population experiences an effect opposite in direction to the intended one, a parallel-group trial reporting a population mean is poorly placed to detect a true effect confined to responders—but the only trial to test patient-level tailoring directly still found no benefit, which is evidence against, not merely a complication of, the responder-selection hypothesis. We regard both propositions as live and unresolved rather than as a settled conclusion, and we return to what would be required to distinguish them in Section 6.2 and Section 7.

### 5.8. Limb-Length Discrepancy

Limb-length discrepancy differs from the preceding conditions in that the mechanical objective is defined geometrically rather than by tissue loading: the aim is to restore symmetry rather than to redistribute pressure. A systematic review has examined shoe lifts for limb-length discrepancy in adults with common painful musculoskeletal conditions [45].

The paediatric evidence is more encouraging than the adult evidence. In children with a mild structural discrepancy, an orthotic insole providing a 50% foot-level correction produced an immediate gait improvement, with an increased step length and velocity and decreased peak plantar pressure in both limbs; significant between-limb differences disappeared for peak ankle dorsiflexion, hip adduction, pelvic upward obliquity, and second peak plantar pressure [46]. The distinction between a heel lift and a full-foot lift is mechanically important, since heel-only elevation places the ankle in relative plantarflexion during the stance and does not correct forefoot height, raising a theoretical risk of plantarflexion contracture over time.

The adult data are less favourable and more revealing. In eight patients with a discrepancy of 2.0 cm or less studied by three-dimensional analysis before and after three weeks of insole use, kinematic adaptations were highly individual; the Dingwell symmetry index showed no significant effect and an unpredictable direction across patients, while a reduction in low back pain appeared to be associated with improved ankle kinematics rather than with a restored symmetry [47]. Related work has demonstrated that limb-length compensation and foot-posture wedging produce measurable changes in proximal muscle activation, with significant reductions in contralateral tensor fasciae latae electromyographic activity under heel-lift compensation [48].

The pattern parallels the knee osteoarthritis literature exactly: the mechanical intervention is delivered, the individual response is heterogeneous and sometimes paradoxical, and the symptomatic benefit that does occur is not mediated by the variable the intervention was designed to correct. In limb-length discrepancy, the clinical implication is that correction should be titrated against a measured gait response rather than prescribed as a fixed fraction of the radiographic discrepancy.

### 5.9. Paediatric Neuromuscular Conditions

In cerebral palsy, the orthotic literature is dominated by ankle–foot orthoses rather than by insoles alone; we include this ankle–foot orthosis evidence because controlled data on insoles alone in this population are scarce, and it should be read as indirect, mechanistically motivated evidence for the sensorimotor and strain-reduction pathways discussed in Section 3, not as direct evidence that an insole reproduces the effects of the ankle–foot orthoses actually studied. With that qualification, the evidence is instructive for the mechanism–outcome question. A systematic review and meta-analysis found strong evidence that ankle–foot orthoses produce small improvements in gait speed, moderate evidence of a small to moderate effect on gross motor function, and strong evidence that posterior devices induce large changes in distal kinematics in children with equinus gait [49]. A scoping review of sagittal-plane gait patterns in spastic bilateral cerebral palsy reached compatible conclusions [50], and a further systematic review compared hinged, solid, floor-reaction, posterior leaf spring, and dynamic designs while noting that most included studies scored poorly on the PEDro scale [51] (Table 4).

The gradient across these findings is itself the finding: the effect is largest on the variable closest to the device (distal kinematics), intermediate on gait speed, and smallest on function. A randomised cross-over pilot measuring community-based walking with ankle accelerometry found no significant group difference in the average daily step count between orthosis-on and orthosis-off conditions [52]—that is, no detectable effect on the outcome furthest from the device.

For paediatric patients in whom an insole rather than an ankle-foot orthosis is appropriate, the patient-specific finite element optimisation described in Section 3.2 offers a route to individualised prescription, having demonstrated in a child with hereditary spastic paraplegia and pes planovalgus that systematic parameter optimisation substantially reduced the peak plantar pressure and improved the load distribution [11].

## 6. Cross-Cutting Themes

### 6.1. The Custom-Versus-Prefabricated Null

The most reproducible finding in this literature is a negative one. A comprehensive review of clinical and cost-effectiveness found no difference between custom-made and prefabricated foot orthoses for pain reduction or functional improvement at six weeks, twelve weeks, or twelve months in adults with plantar heel pain, nor in short-term self-reported recovery or satisfaction, with mixed evidence on comfort [53]. A retrospective analysis of 238 patients found that custom recipients reported a significantly greater PROMIS Physical Function at follow-up (*p* < 0.05) but no difference in pain interference, mobility, or Foot and Ankle SANE scores [54,55]. In the diabetic setting, the custom device achieved measurably better offloading, yet was judged to not be cost-effective [37].

This is not evidence that customisation is mechanically inert—the diabetic data show it is not. It is evidence that the incremental mechanical benefit of customisation, as currently practised, falls below the threshold at which it changes symptoms. Two interpretations follow, and they have opposite implications. Either customisation is genuinely unnecessary for most indications, or the current customisation targets variables that are not the primary mediators of benefit. The pes cavus mechanistic data are consistent with the second interpretation [30,31], though, as noted in Section 5.4, this evidence comes from a single trial’s secondary analysis and should be treated as hypothesis-generating rather than as confirmation that customisation is generally mistargeted across the other nine conditions in this review.

### 6.2. The Responder Problem

Across every condition in which the individual biomechanical response has been examined, the response is heterogeneous and a substantial minority responds in the opposite direction to that intended. The knee osteoarthritis data are the most explicit, with 33% of patients increasing their external knee adduction moment under a lateral wedge [42]; the adult limb-length discrepancy data show the same unpredictability of the symmetry response [47]; and the pes cavus data show that the direction of pressure *redistribution*, not its magnitude, determines benefit [30].

A parallel-group randomised trial in an unselected population containing 33% inverse responders would, in principle, be poorly positioned to detect a clinically meaningful effect confined to the 67% who respond in the intended direction; we present this as a plausible, testable explanation for the field’s null results rather than as an established one, since it has not itself been prospectively confirmed. The most direct available test of this idea—a trial that tailored the wedge angle to each patient’s own biomechanical response before treatment (Section 5.7)—still found no clinical benefit, which weighs against the simplest version of the responder-selection hypothesis and suggests that verified mechanical correction alone may not be sufficient for a clinical benefit even when the target population is, in principle, narrowed. Work identifying the clinically accessible predictors of biomechanical response represents a reasonable next methodological step, but its clinical value, like the responder hypothesis itself, remains to be prospectively demonstrated [43].

### 6.3. Adherence as a Mediator, Not a Covariate

Three independent datasets in this review link the wear time to the outcome: the duration of insole use correlated with pain and function improvement in rheumatoid arthritis [32]; comfort was positively associated with both the wear time and magnitude of pain reduction in a progressive collapsing foot deformity [27]; and full compliance was achieved by only 40% of participants in a diabetic offloading trial [37]. A device worn five hours per day delivers approximately one-third of the mechanical dose of a device worn fifteen hours per day, and no analysis in this literature adjusts for this.

Comfort is therefore an efficacy parameter and should be treated as a primary design constraint. The comfort evaluation of variable-hardness printed orthoses [16] and the participant-preference findings in printed diabetic insoles [38] are appropriately positioned in this respect.

### 6.4. Outcome-Measure Mismatch

The final theme unifies the others. This literature measures peak plantar pressure because it is easy to measure, and measures pain because it is what patients report. The pes cavus re-analysis suggests that, in that specific condition, neither may be the mediating variable: pressure redistribution, quantified through the probability distribution of peak pressure over the gait cycle, predicted pain relief while pressure magnitude did not, in a secondary analysis of one trial [30,31]. We treat this as a hypothesis specific to conditions in which pain relief is the outcome of interest, not as a general critique of peak pressure as a construct—Section 5.6 discusses diabetic ulceration, a condition in which peak local pressure remains an appropriate, mechanistically distinct outcome measure. Whether a distributional variable mediates the outcome in other pain-related conditions is unknown, because it has not been measured.

## 7. A Design-Decision Framework

The practical proposal that follows from this synthesis is to prescribe by the target mechanism rather than by the diagnostic label. Table 5 sets out the resulting framework.

Steps 5 and 6 are the ones absent from current practice and are the ones that the responder data most strongly motivate investigating. A device that does not produce its intended mechanical effect in a given patient is unlikely to produce its intended clinical effect, and continuing to prescribe it risks converting a treatment failure into a chronic non-adherence problem. We present this framework explicitly as a testable, and, in principle, falsifiable, hypothesis rather than as a validated clinical protocol, and we must be direct about two limits on its current standing.

First, we have the feasibility. Steps 2 and 5 as written require an instrumented gait analysis or in-shoe pressure mapping, resources available in specialist biomechanics or gait laboratories but not in most general orthopaedic, primary-care, or community podiatry settings—the same resource gap we identify elsewhere in this review as a barrier to computationally optimised and 3D-printed device adoption (Section 4.3). We did not apply this reality check consistently to our own framework in earlier drafts, and we correct that here: where instrumented verification is unavailable, a resource-limited alternative is to substitute standardised, validated patient-reported outcome measures and a structured fit-and-comfort review at a fixed early interval (for example, two to three weeks) as a proxy for Step 5, accepting that this proxy verifies the tolerability and perceived benefit rather than the specific mechanical target in Step 1. This is a weaker form of verification than gait analysis, and whether it preserves enough of the framework’s intended logic is untested. The costs, equipment, and clinician training implied by the full, instrumented version of the framework are non-trivial and are not analysed in this review.

Second, and more fundamentally, the framework’s only identified direct empirical test argues against, rather than for, the specific mechanism we propose. A trial that tailored the wedge angle to each patient’s individually measured biomechanical response—precisely the logic of Steps 1, 2, and 5 applied to medial knee osteoarthritis—found no significant clinical benefit over a neutral insole (Section 5.7) [44]. We do not think this negative result can be attributed solely to inadequate patient selection without begging the question, since patient-level tailoring against a measured target is exactly what the framework prescribes. A more falsifiable statement of our position is therefore as follows: this framework predicts that, in conditions where a verifiable mechanical target exists, patients whose treatment is confirmed to have achieved that target will show better clinical outcomes than patients whose treatment did not, when compared prospectively within the same trial. This precise comparison has not yet been made for any condition in this review: the one related test available returned a null result, and, until responder-stratified prospective trials of this kind are conducted, the framework should be treated as a structured hypothesis for future study rather than as a recommended clinical pathway.

## 8. Limitations

This review has several important limitations. We discuss them at greater length than is conventional because a number were identified, appropriately, during peer review, and we think readers are better served by an explicit account than by a brief disclaimer.

Depth and breadth of the evidence base. This review draws on 55 references to cover ten clinical conditions together with the mechanistic, manufacturing, and sensor-technology content, which amounts to roughly four to six clinical sources per condition. A dedicated systematic review of a single narrow indication typically synthesises considerably more primary studies than this. We did not perform duplicate independent screening, did not register a protocol, and preferentially selected open-access sources, all of which increase the risk of selection bias relative to a systematic review; screening was performed by one author and checked by the senior author, as stated in Section 2. This review should be read as a cross-condition mechanistic synthesis intended to motivate hypotheses for a future, condition-specific systematic review and prospective study, not as a replacement for such reviews, and readers requiring a definitive evidence appraisal for a single condition should consult the systematic reviews and meta-analyses cited within the relevant subsection.

Scope. We deliberately restricted this review to foot deformity, limb-length discrepancy, and paediatric orthopaedic disease. We did not cover insoles in sports and running biomechanics, in adult neurological populations such as stroke, in balance and fall-prevention applications in the elderly, the independent mechanical contribution of footwear itself as distinct from the insole placed inside it, or a health–economic evaluation of insole provision; each is a substantial work of literature in its own right, and their omission means the mechanistic framework proposed here has not been tested against those bodies of evidence.

Engineering-methods evidence. The finite element and Taguchi-based sensitivity findings summarised in Section 3.2 and Table 2 come from a small number of heterogeneous, largely unvalidated computational studies, at least one of which is a single-patient case report; none pairs its Taguchi analysis with a response-surface or full-factorial interaction check, a limitation discussed in Section 3.2. We treat the resulting geometry-over-material pattern as a hypothesis rather than an established principle, and it should not be read as a clinical effect-size estimate.

Comparator and follow-up limitations in the clinical evidence. Device descriptions are frequently insufficient to reproduce the intervention, comparator insoles described as “sham” or “flat” are mechanically active to an unquantified degree (Section 5.4 and Section 5.5), and follow-up rarely exceeds twelve months in a treatment intended for indefinite use.

Reliance on single-trial secondary analyses. The pressure-redistribution finding in Section 5.4 and the responder-heterogeneity finding in Section 5.7 each rest substantially on analyses of one randomised trial; we have tried throughout this revision to flag both as hypothesis-generating rather than confirmed at the point they are first presented, and we do not consider either established until independently replicated.

The framework’s evidentiary status. The design-decision framework in Section 7 is a synthesis of the evidence above, is presented explicitly as a falsifiable hypothesis, and has one identified direct empirical test, which returned a null clinical result (Section 5.7 and Section 7). It has not been prospectively validated, and should not be applied as a recommended clinical pathway ahead of such validation, and its instrumented steps assume clinical resources—gait analysis, and in-shoe pressure mapping—that are not universally available (Section 7).

Audience and disciplinary scope. Finally, this review deliberately combines clinical–epidemiological and biomechanical–engineering literature and argues for engineering solutions to what is partly a clinical evidence problem. We recognise that this combination creates a risk that a single reviewer’s disciplinary expertise will not span the full manuscript, and we would welcome an evaluation by both a clinical–methodological and a computational–biomechanical reviewer, since we believe both perspectives are necessary to assess the claims made here.

## 9. Conclusions

Insoles reliably do what they are designed to do mechanically and unreliably do what they are prescribed to do clinically. This review has argued that the discrepancy is tractable and is primarily an engineering and measurement problem.

Three hypothesis-generating findings structure the argument, and we restate them here as hypotheses rather than conclusions. Design-parameter sensitivity analyses from a small number of heterogeneous, model-dependent finite element and Taguchi studies consistently identify the arch support geometry as the factor accounting for the largest share of the reported variance in surface pressure and internal tissue strain, with material stiffness contributing more to internal stress; whether this generalises beyond the specific models and parameter ranges examined is untested. The biomechanical response to a given device appears heterogeneous in several conditions, with a substantial minority of patients responding in the direction opposite to that intended, which would, in principle, limit the sensitivity of unselected parallel-group trials—although the one trial that tested patient-level tailoring directly found no clinical benefit, a result that weighs against the simplest version of this hypothesis and is discussed in Section 5.7 and Section 7. And, in the one condition where the question has been asked directly, a secondary analysis of a single trial found that the redistribution of plantar loading, rather than a reduction in its peak magnitude, predicted a symptomatic benefit; if replicated, this would imply that peak plantar pressure is an incomplete outcome measure in that specific pain-related context, a claim we do not extend to conditions such as diabetic offloading, where peak local pressure remains an appropriate proxy for a different, site-specific outcome.

The manufacturing capability required to act on these findings now exists. Digital capture is fast and inexpensive, computational optimisation can explore the design space before fabrication, additive manufacturing can realise continuously varying stiffness fields that conventional construction cannot, and instrumented insoles can verify both mechanical target attainment and wear dose continuously in the patient’s own environment. What is missing is not technology but a prescription paradigm that uses it: one that selects design parameters by target mechanism, verifies that the target was achieved in the individual patient, and abandons the device when it was not. Closing this loop, and prospectively testing whether verified mechanical correction reliably translates into clinical benefit—which the one available direct test of this logic did not confirm—is, in our assessment, a consequential and currently unresolved problem in orthotic bioengineering, and one this review is intended to motivate rather than to claim as solved.

## Figures and Tables

**Table 1:** Mechanisms of insole action, governing design parameters, and verification modality.

Mechanism	Principal Design Parameters	Verification Measurement	Conditions in Which Dominant
Pressure redistribution	Contour conformity, arch height, thickness, material stiffness, apertures, metatarsal addition	In-shoe plantar pressure (peak pressure, pressure–time integral, contact area)	Diabetic neuropathy, pes cavus, metatarsalgia, inflammatory arthritis
Joint moment modulation	Wedge angle, wedge extent, rearfoot posting, device rigidity	3D gait analysis (external knee adduction moment, adduction angular impulse)	Medial knee osteoarthritis, patellofemoral pain
Soft-tissue strain reduction	Arch support height, medial posting inclination, material stiffness	Finite element strain prediction; ultrasound fascia thickness	Plantar fasciopathy, PCFD/PTTD, flexible pes planus
Sensorimotor modulation	Surface texture, top cover, local density variation	EMG timing, postural sway, centre-of-pressure variability	Cerebral palsy, hereditary spastic paraplegia, balance disorders

**Table 3:** Comparison of manufacturing pathways for custom foot orthoses.

Attribute	Plaster/Foam Capture, Thermoforming or Milling	3D Scan, CAD, Additive Manufacturing
Morphological capture	Physical impression; operator dependent	Optical scan; comparable time to traditional [12]
Design freedom	Discrete material zones; contour only	Continuously variable lattice stiffness [13]
Optimisation	Empirical, iterative wear trials	Computational; FE + Taguchi prior to fabrication [7,8,9,10,11]
Reproducibility	Operator dependent	Digital file; batch reproducible
Typical materials	EVA, PORON, Plastazote, polypropylene	TPU, EVA, PP, photopolymer resins [14,15]
Principal limitation	Manual skill, material waste, lead time	Process qualification, comfort validation [16]

**Table 4:** Summary of condition-specific evidence.

Condition	Best Available Evidence	Mechanical Effect	Symptomatic Effect	Ref
Plantar heel pain	Meta-analysis, 19 trials, n = 1660	Consistent	Moderate-quality, medium term; clinical importance uncertain	[2,3,21]
Paediatric flexible pes planus	Systematic review (11 studies); meta-analysis (6 studies, n = 297)	Radiographic angles improved 1.8–5.1°	Uncertain	[4,23]
Adult pes planus/PCFD	Systematic reviews; cohort with adherence data	Reduced pronation, arch flattening	80% improved; comfort predicts wear time	[24,25,26,27]
Pes cavus	Sham-controlled RCT, n = 154	Pressure redistributed across all regions	Pain +8.3, function +9.5 points vs. sham	[28,29,30,31]
Rheumatoid arthritis	Double-blind RCT, n = 80	Forefoot pressure reduction	Pain reduced; correlated with wear time	[32,33]
Juvenile idiopathic arthritis	Multicentre RCT vs. flat sham	—	Pain primary endpoint to 12 months	[34,35,36]
Diabetic neuropathy	Participant-blinded RCT; design meta-analysis	Custom 30% vs. prefabricated 24% PTI reduction	Custom costlier, not superior clinically	[17,37,38,39,40]
Medial knee osteoarthritis	Meta-analyses; tailored-wedge RCT	EKAM reduced ~6%; 33% of patients increase	No superiority over neutral insole	[5,6,41,42,43,44]
Limb-length discrepancy	Systematic review; paediatric and adult gait studies	Paediatric symmetry restored; adult unpredictable	LBP reduction linked to ankle kinematics	[45,46,47,48]
Cerebral palsy	Meta-analysis; scoping review	Large distal kinematic change	Small gait speed, smaller function effect	[49,50,51,52]

**Table 5:** Design-decision framework organised by target mechanism.

Step	Action	Rationale
1. Identify the target mechanism	Pressure redistribution, moment modulation, strain reduction, or sensorimotor input (Table 1)	Determines which design parameters are relevant and which are irrelevant
2. Measure the baseline state	In-shoe pressure map for pressure targets; frontal-plane gait analysis for moment targets	Pressure-informed design approximately doubles achievable offloading [17]
3. Set geometry before material	Arch support height first; posting angle second; heel cup depth for internal stress	Geometry dominates variance in every reported sensitivity analysis [7,8,9,10,11]
4. Select material for internal stress	Softer material with higher arch support minimises fascia strain	Material contributes little to surface pressure but more to stress and strain [7,11]
5. Verify the mechanical target was met	Repeat in-shoe pressure or gait measurement with the device fitted	One-third of knee osteoarthritis patients respond inversely [42]
6. If the target was not met, do not persist	Reconsider mechanism or abandon the device	Persisting with a non-responder generates cost and non-adherence without benefit
7. Optimise for comfort explicitly	Fit review at three weeks; progressive wear schedule	Comfort predicts wear time; wear time predicts benefit [27,32,37]
8. Monitor dose	Instrumented insole or structured wear diary	Only 40% achieve full compliance without monitoring [37]

## Data Availability

No new data were created or analysed in this study. Data sharing is not applicable to this article.
